# FBG-Based Monitoring of Geohazards: Current Status and Trends

**DOI:** 10.3390/s17030452

**Published:** 2017-02-24

**Authors:** Hong-Hu Zhu, Bin Shi, Cheng-Cheng Zhang

**Affiliations:** 1 School of Earth Sciences and Engineering, Nanjing University, Nanjing 210023, China; zhh@nju.edu.cn (H.-H.Z.); zhangchengcheng@gmail.com (C.C.-Z.); 2 Nanjing University High-Tech Institute at Suzhou, Suzhou 215123, China

**Keywords:** fiber optic sensor, fiber Bragg grating (FBG), geological process, geohazard, field monitoring, geotechnical instrumentation

## Abstract

In recent years, natural and anthropogenic geohazards have occured frequently all over the world, and field monitoring is becoming an increasingly important task to mitigate these risks. However, conventional geotechnical instrumentations for monitoring geohazards have a number of weaknesses, such as low accuracy, poor durability, and high sensitivity to environmental interferences. In this aspect, fiber Bragg grating (FBG), as a popular fiber optic sensing technology, has gained an explosive amount of attention. Based on this technology, quasi-distributed sensing systems have been established to perform real-time monitoring and early warning of landslides, debris flows, land subsidence, earth fissures and so on. In this paper, the recent research and development activities of applying FBG systems to monitor different types of geohazards, especially those triggered by human activities, are critically reviewed. The working principles of newly developed FBG sensors are briefly introduced, and their features are summarized. This is followed by a discussion of recent case studies and lessons learned, and some critical problems associated with field implementation of FBG-based monitoring systems. Finally the challenges and future trends in this research area are presented.

## 1. Introduction

Natural disasters occur from time to time all over the world. Earthquakes, tsunamis and volcanic eruptions have caused abundant loss of life and properties in the past few decades. Due to these natural processes, landslides, rockfalls, debris flows, and surface collapses have been triggered. More importantly, under the impact of accelerated industrialization and urbanization, anthropogenic geohazards such as land subsidence and earth fissures in urban areas are growing more serious for communities and infrastructures. The mitigation of these geohazards is a pressing concern for engineering geologists and geotechnical engineers, especially in developing zones and countries.

Geomaterials are natural products of geological processes. Their properties vary continuously in response to geoenvironmental changes and human activities. In order to effectively reduce or prevent the loss of property and human life, real-time monitoring and early warning of potential geohazards are of crucial importance. Since 1930s, geotechnical instrumentation has contributed much to investigate the triggering mechanism of geohazards and to ensure the safety of geotechnical projects. Based on this approach, analytical and numerical geomechanical models were validated, and the effectiveness of stabilizing measures were evaluated. However, as the conventional geotechnical monitoring techniques are normally based on optical, mechanical, hydraulic or electrical sensing elements, there are some inherent limitations [1]. The total station, interferometric synthetic aperture radar (InSAR), and global positioning system (GPS) are widely used ground surface movement detection technologies. They can measure local and regional displacements, but the measuring accuracy is highly dependent on weather and vegetation conditions. In engineering practice, these remote sensing systems are usually used in conjunction with contact monitoring methods. Geotechnical instruments for subsurface movement monitoring, such as inclinometers and extensometers, are incapable of large-scale and long-distance monitoring. For instance, due to the difficulty of arranging data communication cables in the borehole, an in-place inclinometer can hardly measure the slope displacement at a number of depths [2]. Most sensors for measuring earth pressure, pore water pressure, ground temperature, and vibration are point (discrete) sensors. The readings taken in the field are often sensitive to electromagnetic interference (EMI) [3]. The quality of waterproof measures also significantly affects the durability (long-term performance) of these sensors [4]. In order to capture dynamic deformations and evaluate stability conditions of geological structures in real time, it is of great importance to develop an improved smart monitoring system which can overcome the above shortcomings. Only in this way can we forecast geohazard risks and perform early warning with confidence.

In recent years, several fiber optic sensing (FOS) technologies have been developed for monitoring civil infrastructures, such as fiber Bragg grating (FBG), optical time domain reflectometry (OTDR), Raman optical time domain reflectometry (ROTDR), Brillouin optical time domain reflectometry (BOTDR), Brillouin optical time domain analysis (BOTDA), and Brillouin optical frequency domain analysis (BOFDA). Compared with conventional sensors, fiber optic sensors offer a variety of benefits including immunity to EMI, resistance to corrosion, high precision, miniature size and the capability of multiplexing [5]. Among these technologies, the quasi-distributed FBG is one of the most popular and widely used techniques [6]. Using FBG sensing systems, elaborate instrumentation of geohazards can be performed and the detailed information concerning local and overall stability conditions of earth structures can be collected with high reliability.

This paper presents a review of the recent development and applications of the above-mentioned FBG sensing technology in monitoring different types of geohazards, with an emphasis on those triggered by human activities. After a brief background on the working principle of FBG, the development works of applying FBG sensing systems to monitor geohazards are described in detail. Finally, the advantages and limitations of the newly developed FBG sensors are summarized, and some critical issues are addressed. This review is expected to provide valuable insights into FBG-based geohazard monitoring for researchers and practitioners in the discipline of geo-engineering.

## 2. Basics of Fiber Bragg Grating (FBG)

### 2.1. Sensing Principle

The use of optical fibers for digital data communications was proposed by Kao in 1966, and later on he won the 2009 Nobel Prize for Physics. An optical fiber, normally made of glass, is designed to guide light along its length by total internal reflection. With the rapid development of fiber optics, the optical fiber is now dominant in telecommunication, which permits data transmission over longer distances and at higher data rates in comparison with electronic communication. More importantly, the optical fiber can form different types of sensors, which presents unique advantages that have no match in conventional optical, mechanical or electrical-based sensors. When strain or temperature variations occur in an optical fiber, the wavelength or intensity of the scattered, reflected, or transmitted light will change accordingly, which can be measured by an optoelectronic instrument [7]. Depending on the sensor distribution form, the FOS technologies are divided into three types: point sensing, quasi-distributed sensing, and fully-distributed sensing. The sensors can be further categorized into several types in terms of sensing principle, e.g., intensity based, reflection based, interferometry based, and scattering based. FBG is a popular quasi-distributed FOS technology, which makes use of a special phenomenon of the reflected light due to Bragg grating in an optical fiber.

**Figure 1:**
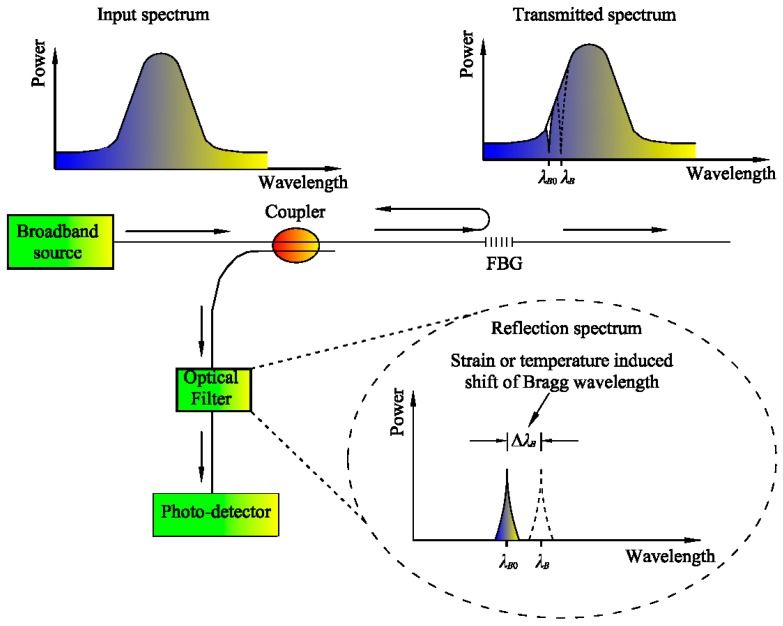
Generic concept of the FBG sensor.

The physical fundamentals for FBG were set by W. H. Bragg and his son W. L. Bragg, for which they both won the 1915 Nobel Prize. The first FBG sensor was fabricated by Hill et al., a scientist at the Communications Research Centre in Ottawa, Canada [8], who discovered for the first time the photosensitivity phenomenon in Ge-doped silica fibers. The Bragg grating can be written into a segment of Ge-doped single-mode optical fiber using a spatial pattern of ultraviolet (UV) light. Figure 1 illustrates the working principle of an FBG sensor. According to Bragg’s law, when a broadband source of light has been injected into the fiber, FBG reflects a narrow spectral part of light at a certain wavelength [9]:

(1) $$\lambda_{B}=2n_{eff}\Lambda$$

where $\lambda_{B}$ is called the Bragg wavelength; $n_{eff}$ is the effective core index of refraction; Λ is the periodicity of the index modulation.

The Bragg wavelength is strain- and temperature-dependent through physical elongation or thermal change of the sensor and through the change in the fiber refractive index due to photo-elastic and thermo-optic effects. Considering a standard single mode silica fiber, a change in strain Δε or temperature ΔT will alter the Bragg wavelength $\lambda_{B}$ as follows [10]:

(2) $$\Delta \lambda_{B}=2n_{eff}\Lambda \left[\left(1-\frac{n_{eff}{}^{2}}{2}\right)\left[p_{12}-\nu \left(p_{11}-p_{12}\right)\right]\Delta \varepsilon +\left(\alpha +\frac{\frac{dn_{eff}}{d\Delta T}}{n_{eff}}\right)\Delta T\right]$$

where $\Delta \lambda_{B}$ is the change in Bragg wavelength due to applied strain and temperature change; $p_{ij}$ (*i*, *j* = 1 or 2) are the Pockel’s coefficients of the stress-optic tensor; ν is Poisson’s ratio; α is the coefficient of thermal expansion of the fiber.

Equation (2) can be simplified as:

(3) $$\frac{\Delta \lambda_{B}}{\lambda_{B0}}=\left(1-p_{eff}\right)\Delta \varepsilon +\left(\alpha +\xi \right)\Delta T=c_{\varepsilon }\Delta \varepsilon +c_{T}\Delta T$$

where $\lambda_{B0}$ is the original Bragg wavelength under strain free and 0 °C condition; ξ is the thermo-optic coefficient; $c_{\varepsilon }$ and $c_{T}$ are the calibration coefficients of strain and temperature; $p_{eff}$ is the photo-elastic parameter defined by the following equation:

(4) $$p_{eff}=\frac{n_{eff}{}^{2}}{2}\left[p_{12}-\nu \left(p_{11}-p_{12}\right)\right]$$

In 1993, Hill et al. proposed a cost-effective method for fabricating FBG using the phase mask, which makes mass production possible [11]. Since then, FBG has been widely used in a variety of fields all over the world. By far, it is one of the most popular FOS technologies for health monitoring of civil infrastructures. A main reason for this popularity is that, with special configurations, FBG can monitor many physical parameters, such as temperature, strain, relative displacement, humidity, and pressure, offering excellent measuring resolution and range, absolute measurement, and modest cost per channel. As the sampling rate of FBG is up to 2 MHz, it is suited for both static and dynamic measurement. Furthermore, because FBGs are passive sensors, they can be either time- or wavelength-multiplexed, which allows for quasi-distributed sensing. Owing to these advantages, FBG is very attractive for geohazard monitoring.

### 2.2. Demodulation and Multiplexing Schemes

According to the sensing principle, FBG can be used as a strain sensor or a temperature sensor. To measure wavelength shifts that result from variations in temperature or strain, an FBG sensing system should have an optical source that continuously interrogates the reflection spectrum, and a detection module that records the shifts in the peak reflectivity versus wavelength. Several interrogation configurations have been developed for reading out the Bragg wavelength shifts experienced by FBG, from which strain and temperature are calculated.

**Figure 2:**
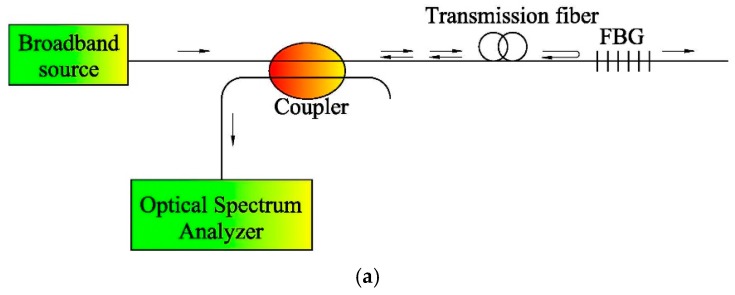

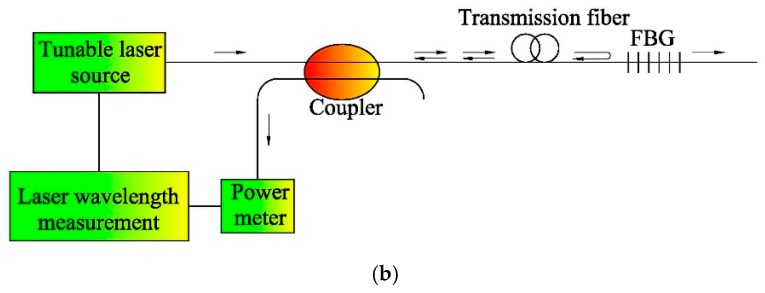
Typical FBG interrogation configurations [12]. (**a**) FBG measurement with a broadband source and an OSA; (**b**) FBG measurement with tunable laser and reflected power detection.

Primarily, there are two techniques for detecting the precise wavelength of the peak in the FBG reflected spectrum. The first method, as shown in Figure 2a, is to send light from a broadband source into the optical fiber and analyze the spectrum of the reflected light. In this system, an optical circulator is used to transmit the outgoing light into the transmission fiber and redirects the back-reflected light to an optical spectrum analyzer (OSA). Michelson interferometry-based OSA can provide most accurate wavelength measurements [10]. This method has been used for many years in wavelength meters to determine the wavelength of laser sources with a high degree of accuracy.

The second method is based on a narrowband tunable laser source, as shown in Figure 2b. The tunable laser source sweeps over the spectral range including the FBG spectral peak. When a peak in the reflected intensity is detected by a power meter, the measurement module of laser wavelength records the accurate wavelength of the scanning laser. Using this configuration, the measuring accuracy of wavelength can be as high as 1 pm (1 pm = 10^−12^ m) or, equivalently, less than 1 με or 0.1 °C [12].

A most important attribute of FBG is that the sensors can be multiplexed in parallel or in series [12]. In this way, a quasi-distributed strain or temperature sensing array can be established. Currently, there are two mature schemes to interrogate such a string of sensors. The first one is called time division multiplexing (TDM), in which the time of flight using short duration light pulses is used to discern which signal is reflected from which grating along the fiber path. In theory, over 100 FBG sensors with the same Bragg wavelength can be on an optical fiber if the sensor spacing is large enough.

The second and most utilized approach is wavelength division multiplexing (WDM). This approach requires all the serially connected FBG sensors should pose sufficient spacing of the Bragg wavelengths (e.g., 10 nm = 10^−8^ m) to avoid spectral overlap during measurement. The interrogator system uses these unique Bragg wavelengths to keep track of the sensor locations. Sensor capacity on each optical fiber is dependent on the measurement range of each FBG sensor and the total spectral range of the interrogator. The typical range of a commercially available interrogator is 1510 to 1590 nm or 1525 to 1565 nm. Sometimes, WDM and TDM can be combined to form an FBG sensing network.

Recently, Kung et al. introduced an FDM technology, which can simplify the set up and installation of complex WDM scheme [13]. This method requires a tunable laser and limited number of sensors because of wavelength constraint in the communication band.

### 2.3. Temperature Compensation

According to the sensing principle of FBG, strain and temperature changes directly affect the period of the index modulation as well as the effective index of refraction of FBG. It is noted that monitoring of geohazards are usually long-term tasks. Any change in environmental temperature alters the Bragg wavelength and consequently the measured strain data, especially for cases where large daily and seasonal temperature differences are encountered. As a consequence, temperature compensation is an inherent task for a FBG field monitoring system. In this area, two methods are frequently adopted, namely direct and indirect ones [14].

A standard FBG sensor cannot possibly measure strain and temperature simultaneously. If the temperature variation ΔT is captured using thermocouples, resistance thermometer detectors (RTDs) or ROTDR fibers, the strain measurement of an FBG sensor can be temperature compensated using the following equation [15]:

(5) $$\Delta \varepsilon =\frac{1}{c_{\varepsilon }}\left(\frac{\Delta \lambda_{B}}{\lambda_{B}}-c_{T}\Delta T\right)$$

Alternatively, the indirect method of temperature compensation utilizes an additional FBG sensor that is installed in the same sensing region to compensate for the thermal induced strain [16,17]. The thermal compensation sensor should be free of any mechanical strain, so a loose-buffered optical fiber where the core, cladding and coating can move freely inside the jacket, making this sensor immune to the deformation of soil or rock. Calibration should be made prior to field implementation. The discrimination of the wavelength shifts due to strain and temperature variations, i.e., $\Delta \lambda_{B}{}^{\varepsilon }$ and $\Delta \lambda_{B}{}^{T}$, can be achieved. Consequently, the corrected strain measurement is obtained as follow [17]:

(6) $$\Delta \varepsilon =\frac{\Delta \lambda_{B}{}^{\varepsilon }}{c_{\varepsilon }\lambda_{B}}=\frac{\Delta \lambda_{B}-\Delta \lambda_{B}{}^{T}}{c_{\varepsilon }\lambda_{B}}$$

## 3. Development of FBG-Based Geohazard Monitoring Systems

As mentioned before, geohazard monitoring faces myriad challenges. In the past two decades, a number of FBG sensors and systems have been developed for providing valuable and timely information related to geohazards. Using quasi-distributed FBG sensors, the variations of temperatures, displacements, loads, earth pressures, pore water pressures and soil moistures can be captured with high accuracy.

### 3.1. Installation and Protection Techniques

One of the toughest tasks in utilizing FBG sensing system for field monitoring is how to install the sensors and protect them from physical damage. As FBG has inherent vulnerability, bare FBG sensors can hardly be applied to monitoring infrastructures without appropriate packaging methods [18]. Once a suitable protection measure is adopted, the FBG sensors should be calibrated, because any protection technique may affect the strain transfer efficiency from the fiber surface to the inner core [19,20]. At present, until methods and standards for installing fiber optic sensors in the field are established and reliable, close collaboration of experienced geo-engineers and skilled optoelectronic engineers is necessary [21].

Existing packaging methods include gluing FBG to the target and embedding with adhesives [22,23,24,25,26], packaging FBG in pipes or on plates [25,27,28,29,30], or encapsulating FBG into composites or layered structures [20,31,32,33,34]. For field geohazard monitoring, the installation works can be extremely tricky since the optical fibers can hardly be adhered on geomaterials. Numerous studies have demonstrated the high adaptability of fiber–reinforced polymer (FRP) to FBG sensors. Therefore, it is wise to use FRP bars, tubes, of strips to make a sensor protection system in the field.

Although the soil-embedded FBG sensors can capture the strain variations at different locations of geological structures, it is hard to directly bury them into in-situ soil masses. A common practice is to adhere the sensors on- or embed them in-soil reinforcements, such as geogrids, soil nails and anti-slide piles. As early as 1999, the use of glass fiber–reinforced polymer (GFRP) rock bolts with integrated FBG sensors for strain monitoring in a Switzerland tunnel was reported by Frank et al. of the Swiss Federal Laboratories for Materials Testing and Research [31]. Nellen et al. successfully monitored strain and temperature fields of tunnels with FBG instrumented GFRP rock bolts [35]. Briancon et al. investigated the feasibility of smart geotechnical monitoring using FBG embedded in geotextiles [36]. Later on, the FBG sensors were substituted by fully distributed BOFDA fibers to make intelligent geosynthetics [24,37]. Three optical fiber cables instrumented with an array of low reflectivity fiber gratings, with the same Bragg wavelength, were stitched onto a 1-m high geotextile by Kung et al. [13]. The geotextile was not only capable of detecting minute deformation, but also provided a large resisting force against the landslide. Wang et al. developed a smart geogrid embedded with FBG strain sensors [38,39]. The sensor features were evaluated through finite element simulation and lab model tests.

In the field study of Zhu et al. [25], painstaking precautions were taken to waterproof conventional strain gauges installed on GFRP soil nails to ensure their survivability. In comparison, there was no such need for FBG sensors but how to protect the FBG sensors from physical damage was difficult. In their field work, when the workers tried to insert the soil nails in the boreholes, some surface-adhered FBG sensors were damaged.

Because of the complicated geological conditions and harsh installation processes, optical fiber cables and connectors used for transmitting monitoring data have to be well protected to prevent physical damage or signal loss. No-jacket-buffered optical fibers should be avoided for direct embedding into geomaterials. To reduce errors in obtained monitoring data, the coating of the cable should be correctly selected according to different site conditions and measurement requirements. Metals and plastics are two commonly used buffering materials. For geohazard monitoring, the cable diameter can vary from 0.25 to 10 mm. Iten et al. declared that it is sufficient to adopt optical fiber cables with less than 2 mm diameter for an experienced geo-practitioner when performing manual integration [40].

In Canada, Huntley et al. conducted real-time monitoring of the Ripley Landslide using FBG and BOTDR sensors [41,42]. They stated that, after three months’ monitoring, their field instrumentation works stopped as the optical fiber cables were damaged by black bear activity. Epoxy resin and caulking products also failed during prolonged intervals of sub-zero temperatures, and the cables could not be firmly attached to the retaining wall. Their experiences indicate that periodic on-site visits are recommended to trouble-shoot hardware, software and telemetry issues.

### 3.2. Development of FBG Temperature Sensors

**Figure 3:**
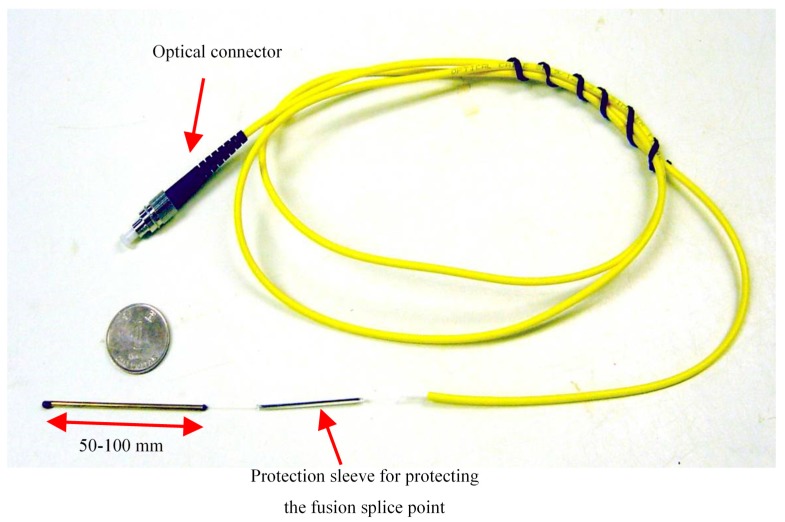
Photograph of the tube packaged FBG temperature sensor [12].

In the past two decades, different types of FBG sensors have been developed for geohazard monitoring. The simplest sensor is the FBG temperature sensors. A commonly used FBG temperature sensor consists of a loose FBG sensor encapsulated in a tube (Figure 3). When applied in the field, this sensor is mechanically uncoupled from but in thermal contact with the host material. In general, the temperature resolution is 0.1 °C or less. Dewynter et al. installed FBG thermal probes in a borehole surrounding a central heating borehole to conduct in-situ thermal measurements of argillaceous rocks [43]. The FBG results were in good agreement with those from conventional resistive probes. Brambilla et al. pointed out that gratings written in tin-doped silica fibers exhibited great thermal stability up to about 800 °C, surpassing those written in borogermanosilicate, germanosilicate or hydrogen loaded telecom fibers, which were potentially suitable for high temperature applications such as in volcanic areas [44].

### 3.3. Development of FBG Displacment Sensors

Monitoring of large strain and displacement is a crucial part for geohazard monitoring. Although the FBG sensors developed for displacement monitoring generally have satisfactory accuracy owing to the intrinsic characteristics of the sensing principle, the measuring range is a bottleneck problem. The ultimate strain of silica optical fibers is merely around 5% [45], which significantly limits the wide application of this technology. The polymer optical fiber (POF) can overcome this problem, since its ultimate strain is about 40% [24]. However, FBG can only be fabricated on a silica fiber.

Recently, some FBG sensors are developed for displacement monitoring in boreholes. Schmidt-Hattenberger and Borm designed an FBG extensometer to monitor rock deformations in tunnels, which was embedded in GFRP rock bolts [46]. Zhu and Yin introduced the application of FBG settlement tubes to measure the vertical profile of settlement in boreholes under a mat foundation [12]. In this sensor system, a pre-tensioned FBG is packaged in a plastic tube, which is connected to a stainless steel spring. The measured strain *ε* is thus proportional to the tube compression *S*, i.e., [12]:

(7) $$S=\left(\frac{\pi dtE}{k}+l\right)\varepsilon =\left(\frac{\pi dtE}{k}+l\right)\frac{\left(\Delta \lambda_{B}-\Delta \lambda_{B}{}^{T}\right)}{c_{\varepsilon }\lambda_{B0}}$$

where *E*, *d*, *l* and *t* are the Young’s modulus, outer diameter, length and thickness of the tube, respectively; *k* is the spring coefficient; $\Delta \lambda_{B}$ and $\Delta \lambda_{B}{}^{T}$ are the shifts in Bragg wavelength of the FBG strain sensor and an FBG temperature compensation sensor.

**Figure 4:**
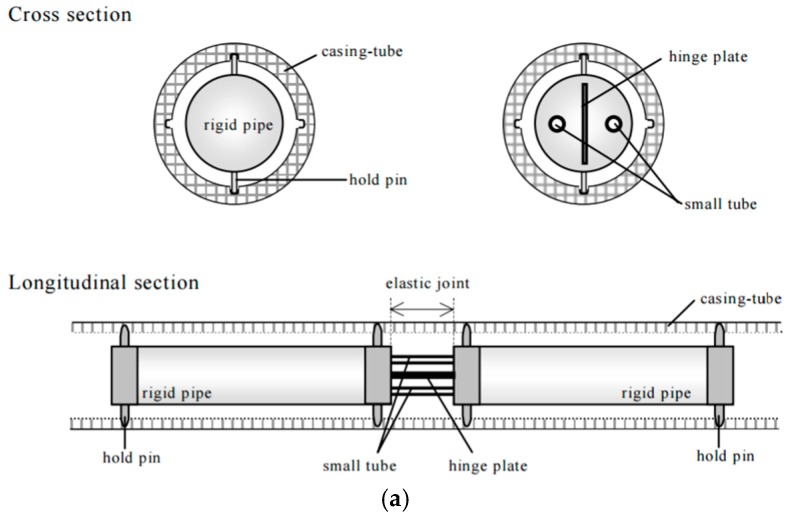

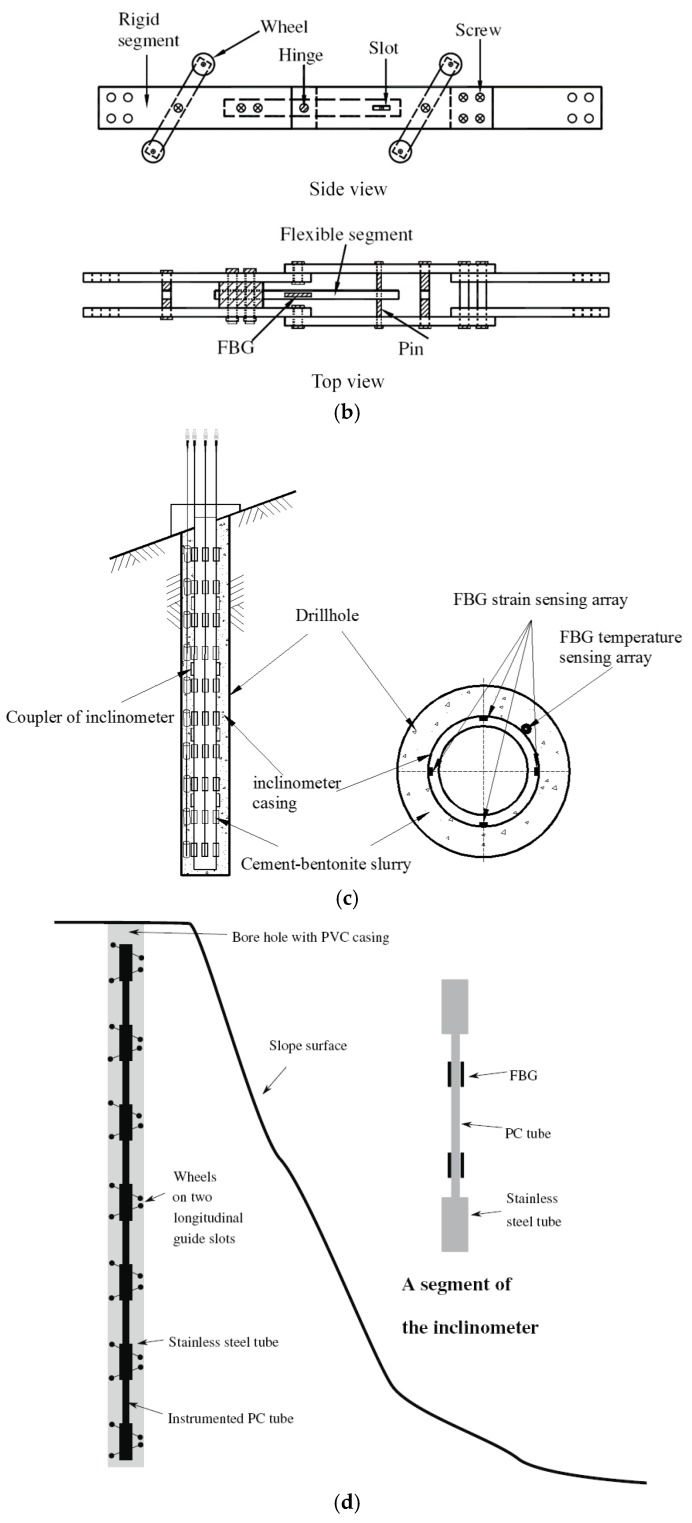
Schematic illustration of typical structures of FBG-based inclinometers. (**a**) FBG borehole inclinometer [47]; (**b**) FBG segmented deflectometer [16]; (**c**) FBG in-place inclinometer [15]; (**d**) New-generation FBG in-place inclinometer [45].

The conventional inclinometer has been widely utilized to monitor slope displacements and ground movements. However, long-term use of inclinometers is impractical due to the disadvantages of low accuracy, poor durability, complex operation, and high costs. In recent years, several studies of in-place slope displacement monitoring using FBG-based techniques have been reported [15,16,47,48,49,50]. Figure 4 shows the typical structures of FBG in-place inclinometers. The combination of conventional inclinometers and the FBG technology effectively improves the measurement range of displacement.

In Japan, Yoshida et al. developed the first generation of FBG-based borehole inclinometer for slope deformation monitoring following the principle of the conventional deflectometer [47] (Figure 4a). This type of inclinometer is composed of a series of rigid pipes connected to each other with elastic joints. Bending of the joints is measured by FBGs embedded on the elastic joints. The inclinometer was installed in boreholes for deformation monitoring of an artificial slope under construction. Late on, a borehole multiple deformation sensing system was developed by Kashiwai et al. using internal FBG sensor units [2]. When ground displacement occurs, the FBG-instrumented plate element will bend accordingly and the FBG readings can be self-temperature compensated. This sensing system was successfully used for ground deformation monitoring of a tunnel in Japan.

Another FBG segmented deflectometer was invented by Ho et al. [16]. The deflectometer is designed to be inserted into the conventional inclinometer casing installed in vertical boreholes to measure the relative deflection between the segments of the inclinometer casing (Figure 4b). Laboratory and field experiments were conducted to verify the effectiveness of the FBG system. The test results were consistent with those of a conventional inclinometer probe, indicating that the system could be used for ground movement monitoring. However, the measurement error is amplified with distance due to the computation method, which may limit the effective length of the deflectometer in field use.

Zhu et al. reported the fabrication of a polyvinyl chloride (PVC) casing with FBG strain and temperature sensors adhered to the grooves of its surface [15] (Figure 4c). The integral method was utilized to calculate the displacements in different depths based on linear interpolation of strain distribution along the PVC casing. Wang et al. successfully installed this inclinometer in nine boreholes for landslide monitoring in Wenzhou, China [50]. The slip surface determined by the monitoring results fit well with that calculated by limit equilibrium method. In Europe, Habel et al. used a similar approach to monitor a sliding coal pit slope [21]. In a shaking table test, Xu et al. employed the same method to calculate the lateral displacements of a small bar buried in soil [51]. The shortcomings of this inclinometer are that the boundary conditions have to be correctly specified and the measuring errors will accumulate during numerical integrations.

As a very significant continuation of this work, a new generation of FBG-based in-place inclinometer was developed by Pei et al. [49]. To make an inclinometer, the FBG-instrumented elastic bars were connected to stiff steel tubes equipped with sliding wheels. Then they are installed in a borehole through inclinometer casings. The FBG sensors can measure two parameters, i.e., the relative deflection and rotation angle of two ends of every bar. The displacement profile with respect to depth can be calculated using the superposition approach. As shown in Figure 4d, based on the classical indeterminate beam theory, the deformation of segments is coordinated with the casings and accumulated error in the displacement superposition process is efficiently avoided. Another benefit of this inclinometer is that the displacement measuring range can be enhanced using closely spaced sensing segments. Through field applications, the performance of this in-place inclinometer was verified.

### 3.4. Development of FBG Pressure Sensors

**Figure 5:**
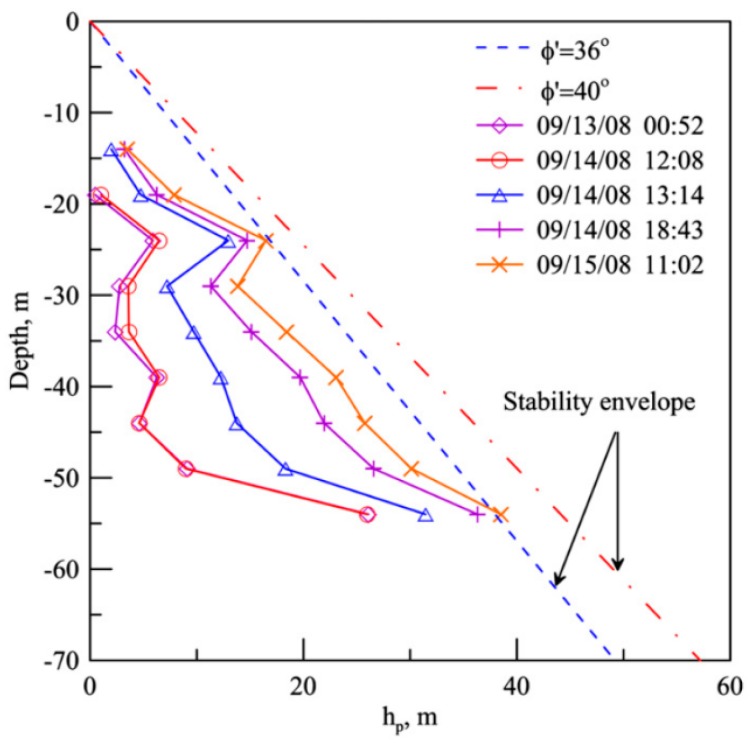
Profile of *h_p_* during typhoon Morakot [55].

Besides displacement sensors, FBG can be pressure sensors for measuring earth pressure or pore water pressure. Chang et al. developed innovative pressure transducers using FBG strain sensors [52]. They were used for performance evaluation of pavements and weigh-in-motion measurement. Three methods for bonding the FBG to the transducer diaphragm were considered. Owing to the disc shape of the bulkhead, the FBG sensor is designed to be installed circumferentially so that the optical spectrum of the sensor will show a clear peak. A self-temperature compensated chirped FBG pressure sensor was developed by Ho et al. [53]. They demonstrate through laboratory calibrations that this sensor can perform timely and accurate measurement of pore water pressure over a wide range of temperatures. On the basis of indirect sensing using epoxy resin-embedded FBG sensors, Correia et al. developed a pressure sensor with a resolution of 0.66 kPa over a measurement range of 300 kPa, which was suitable for measuring pore water pressures in soil [54]. A similar sensor, but instead encapsulated in a softer material of high Poisson’s ratio, was devised by Legge et al. with the advantage of enhancing the longitudinal strain induced in the fiber [4]. Huang et al. measured the pore pressure profile in boreholes using FBG sensors and evaluated the stability of rainfall-induced deep landslides [55] (Figure 5). Wu et al. introduced the application of FBG mini-osmometer for measuring the pore water pressure of the main pumping aquifer in the field [56]. Compared with piezometers, the FBG sensors can be serially connected to capture the distribution of pore water pressure with respect to depth.

### 3.5. Development of FBG Sensors for Other Applications

In addition to temperature, strain, displacement and pressure, FBG sensors can also be applied to measure other physical parameters such as humidity and moisture. The reviews by Yeo et al. [57] and Alwis et al. [58] detailed the recent development of this kind of sensor, and concluded that such concept is still fairly new. While interesting works have been reported on the use of FBG sensors for soil moisture measurement [59,60], its applications in geohazard monitoring have still been rare. It is noteworthy that, using ROTDR or BOTDA technologies, distributed temperature sensing (DTS) optical fiber cables have been successfully applied to monitor soil moisture or seepage [61,62,63].

Another potential of FBG sensors is to measure ground vibrations generated by earthquakes, landslides, debris flows, rock falls, sinkhole collapse, etc. An important parameter to consider when devising FBG accelerometers is the operating frequency range, which varies with different situations. Huang et al. developed a fiber-optical sensing system comprising commercial FBG accelerators (operating frequency range: 0–300 Hz) for debris flow monitoring [64]. Field monitoring data were compared with conventional geophones, showing that this system was feasible for debris flow detection. The seismic waves induced by earthquakes have a much broader range, from 10^−4^ Hz up to 10^2^ Hz. Achieving such broad range for accelerometers, seismometer networks, or FBG-based sensors is still a tough job. An FBG seismic accelerometer was developed by Gagliardi et al. using a flexural cylinder beam [65]. This accelerometer can detect signals of a maximum frequency of 30 Hz, a maximum acceleration of 2.5 *g*, and a dynamics of 100 dB. Such parameters were declared to meet most requirements for a seismic sensor.

## 4. Current Status of Applying FBG Systems for Geohazard Monitoring

**Figure 6:**
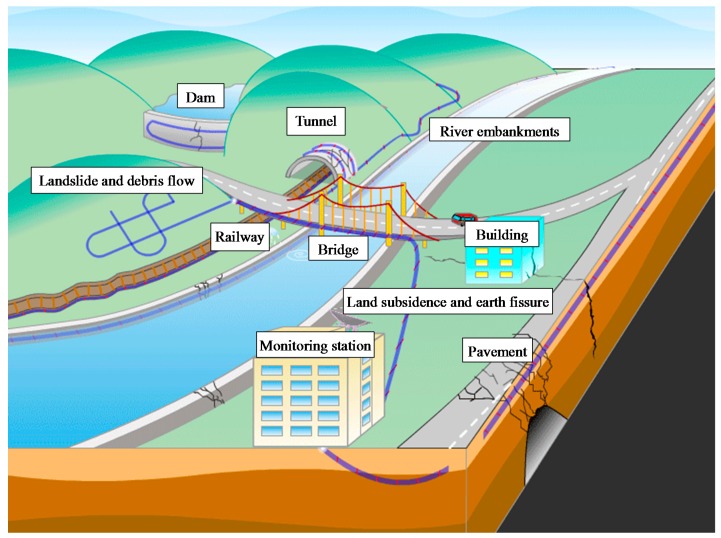
Schematic illustration of geohazards and infrastructure monitoring using FOS technologies.

In the past two decades, numerous investigations have been conducted to monitor different types of geohazards all over the world. Figure 6 illustrates the use of FOS systems in geohazards and infrastructure monitoring. Some case studies related to FBG are presented and discussed below.

### 4.1. Slope Stability and Landslide Monitoring

#### 4.1.1. Performance Monitoring of Slope Reinforcements

A number of slope stabilization methods have been used in engineering practices, such as geosynthetics, anchors, soil nails, side-resistant piles, and retaining walls. As previously mentioned, the bare FBG sensors are fragile in strength. Due to the magnitude of potential geohazards and chaotic nature of construction sites, the sensors are easily damaged. If they are installed on the slope reinforcing members, adequate protection of the sensors can be realized. More importantly, the FBG measurements can help evaluate the slope stability condition and even forecast potential disasters. Comparing with electrical resistance and vibrating-wire strain gauges, FBG sensors can be quasi-distributed along the slope reinforcements, so that the distribution of axial force, shear force and bending moment can be obtained.

Schroeck et al. [66] and Willsch et al. [67] introduced the use of FBG sensors for mine monitoring in Germany. For an anchor bolt, the strain sensing fiber was glued in a groove and covered by special epoxy resin, with a fiber for temperature compensation loosely laid on the other side of the anchor bolt. They suggested to install the sensing fiber on the reinforcing steel bars inside anti-sliding piles, shaped in the form of the letter U. Monitoring of slope displacements could then be made. Another case was reported by Dou and Li [68], who employed 50 FBG-instrumented anchor bolts to monitor the Xiaolongtan slope in Yunnan, China.

Zhu et al. successfully monitored strain distributions along soil nails and soldier piles using FBG sensing systems in Hong Kong [15,25]. They evaluated the performance of two GFRP soil nails during field pullout tests based on the monitoring results of quasi-distributed FBG sensor arrays [25]. The surface-glued and tube-packaged FBG sensors were installed along the soil nails for axial strain monitoring, together with several strain gauges. The monitoring results show that, compared with strain gauges, the FBG sensors can perform accurate strain measurement on multiple locations, which clearly depicted an approximately linear strain distribution with respect to the nail length during the pullout tests. As the observed maximum strain was over 7000 microstrain, the GFRP soil nail was found to be a highly extensible soil reinforcement, which requires additional displacement-based criteria to limit the allowable pullout resistance.

**Figure 7:**
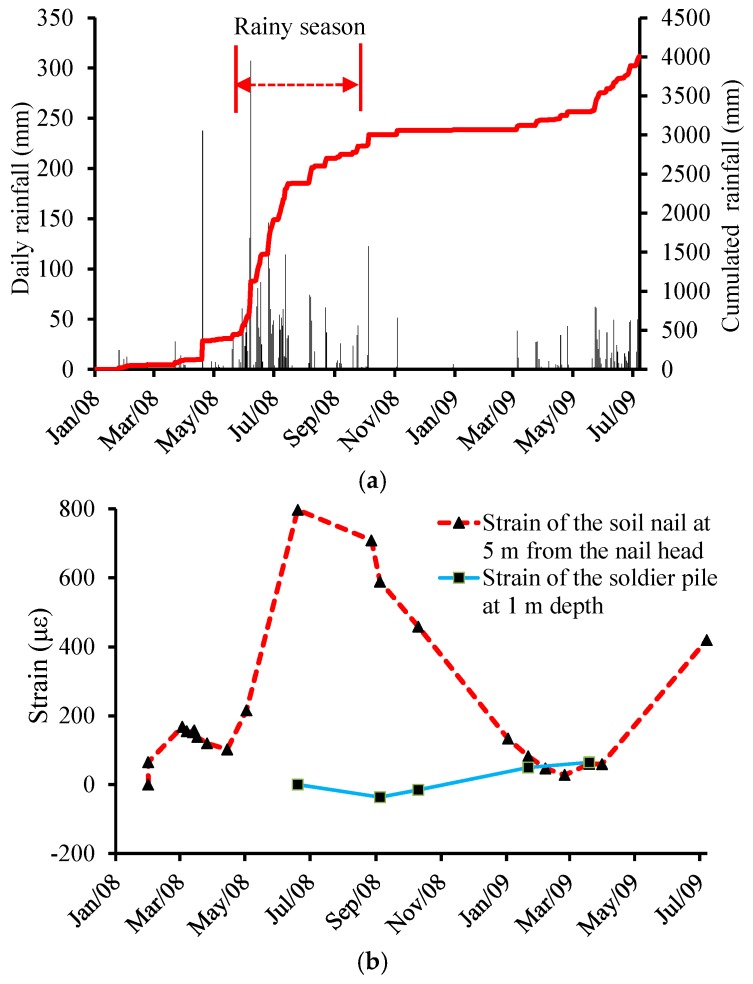
Long-term monitoring results of the slope site in Hong Kong (adapted from [15]). (**a**) Rainfall; (**b**) Strains of the soil nail and the soldier pile.

On the Luk Keng roadside slope when upgrading works were being implemented, a 14 m-long soil nail was instrumented with 10 FBG strain sensors and 10 corresponding FBG tube packaged temperature sensors [15]. The soil nail was then inserted into the drillhole and grouted with cement grout. An H-shaped steel column instrumented with 18 FBG strain sensors and nine temperature sensors was grouted in a 16 m-long borehole to form a soldier pile. During installation, PVC trunkings were used to protect the optical fiber cables. The strain variations in the soil nail and the soldier pile with monthly rainfall were compared to each other (see Figure 7). It was seen that the soil nail strains were in general consistent with monthly rainfall, but such a relationship was not found for the soldier pile, which was not been clearly documented in previous works.

Huntley et al. installed an integrated fiber optic sensing system to monitor the performance of a lock-block retaining wall on the Ripley Landslide in Canada [41,42]. This is a retrogressive translational landslide that have been active since 1951. Two FBG sensors were fixed on the retaining wall, together with surface-glued BOTDR cables. Three months of monitoring data show there was no obvious displacement, indicating the retaining wall was stable.

#### 4.1.2. Monitoring of Slope Movements

Conventionally, slope movements monitoring is performed using inclinometers, extensometers, time domain reflectometry (TDR) cables, tiltmeters, and geodetic photogrammetry techniques such as GPS and InSAR. The limitations of these methods have been mentioned previously. Recently, both surface and subsurface FBG-based slope deformation monitoring have been reported. Moore et al. installed a long gauge FBG sensing system in southern Switzerland for continuous rockslide monitoring [48]. There were two types of sensors, i.e., fully embedded borehole sensors and surface extensometers. The monitoring system detected sub-micrometer scale deformations, which offers new insight into the deformation process of the test site. A similar field trial was carried out by Zalesky et al. in Czech. In their study, the performance of the FBG sensors was compared with the fully distributed BOTDA sensors [34].

**Figure 8:**
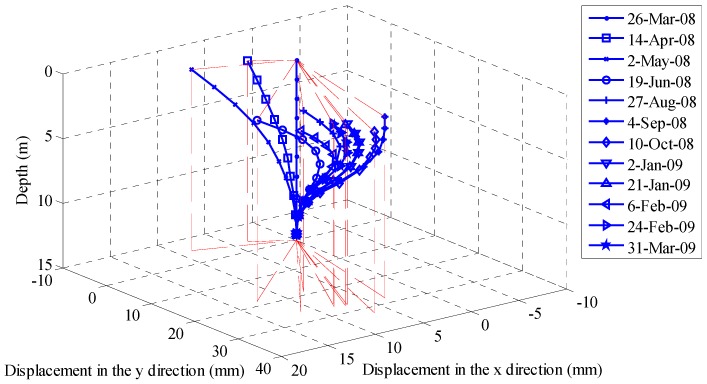
Monitoring results of slope movements (adapted from [15]).

Ma et al. used FBG-instrumented strain tubes to monitor the subsurface displacement of Erlangmiao landslide crossing oil and gas pipelines in China [69]. The measured results indicate that this was a shallow landslide. Zhu et al. reported the monitoring of subsurface deformation at the Luk Keng slope using newly developed FBG in-place inclinometers [15]. As shown in Figure 8, the slope movements developed in a three-dimensional pattern, due to the orientation of weak zones within the slope mass.

With reliable installation method, FBG sensors can perform direct measurement of soil strain. In this area, some preliminary investigations have been conducted by Zhu et al. [17,70]. They carried out a series of laboratory model tests on slope stability. Quasi-distributed FBG strain sensors were directly embedded in the slope models in the horizontal and vertical directions. The soil strains during surcharge loading and groundwater level variations were measured, which provides critical insights into the slope deformation and failure modes.

#### 4.1.3. Forecasting and Early Warning of Landslides

Landslide warning is of great significance in mountainous regions where people live directly located below slopes. Time-estimation and location-determination of an impending collapse are two fundamental parts of a landslide prediction program. As pointed out by Kato et al. for a road situated below a potential landslide, a sensing system should be able to predict the landslide at least 1 to 2 h in advance of the occurrence [7]. They reported an FOS system for displacement monitoring and collapse prediction in several landslide sites in Japan. In these studies, the FBG technology was combined with BOTDR and Macro Distortion Monitor (MDM). The results show that the FBG sensors detected the abnormal strain variations 2 h before the slope collapsed.

In China, Zhang et al. investigated the feasibility of predicting landslides based on the monitoring data of FBG-instrumented rods [71]. Soft and hard rods were buried in the slope mass and an artificial landslide was triggered by manual excavation. The test results indicate that, to make the measurements sensitive, it is important to optimize the installation locations of the sensors.

The setting of appropriate warning and alarm threshold values is another open issue for landslides warning. The development of accurate FBG systems makes it possbile to sense minute deformations. However, it is still a challenge to establish a forecasting model and to determine alarm trigger conditions based on the FBG monitoring results. In this aspect, a pilot study was conducted by Zhu et al. [72]. Through physical and numerical modeling, they investigated the feasibility of slope stability evaluation for locally loaded slopes based on strain measurements. It is found that the strain distribution characteristics captured by FBG sensors are closely related to the propagation of plastic zones and the formation of critical slip surfaces. Taking into consideration the convenience of field instrumentation and monitoring sensitivity, the maximum strains at multiple elevations can be used as characteristic parameters for setting landslide warning threshold values.

### 4.2. Debris Flow Monitoring

**Figure 9:**
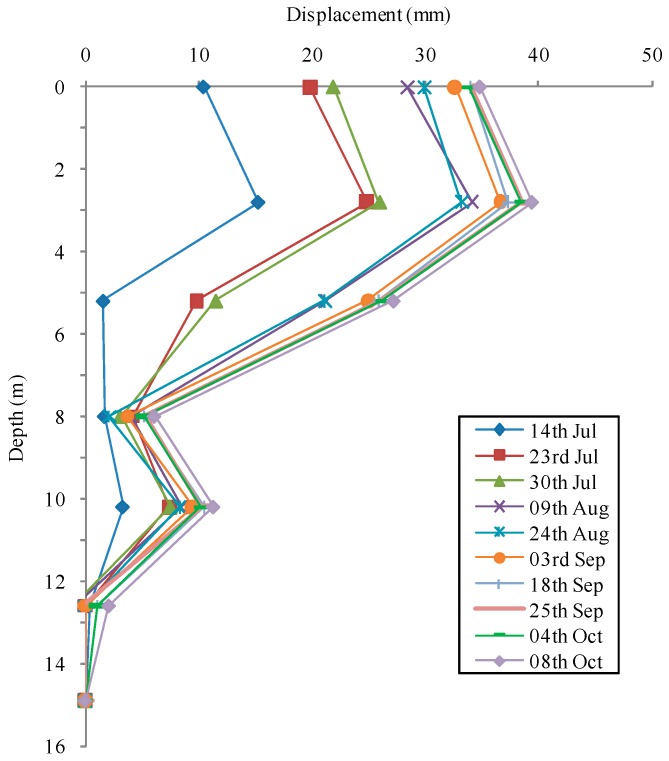
Borehole displacements at the lower part of the debris flow site [49].

The conventional instruments for debris flow monitoring include photocells, geophones, seismometers, and wire, ultrasonic, laser and radar sensors [73]. The key problem here is how to detect the initiation of the debris flow. Monitoring and warning of debris flows using FBG inclinometers and column-nets was reported by Pei et al. [49]. The inclinometer can perform real-time measurement of borehole displacements, as seen in Figure 9. The FBG column-net, with FBG sensors glued on the bottom of its steel pipes, was installed in the debris flow direction and functioned by detecting the strain at the bottom, which was affected by impact forces when debris flows pass by. In Weijiagou Valley, southwest China, the FBG sensors were used to detect the initiation of debris flow process. Once the impact force rises up to a certain value, the column-net will break. The researchers, however, did not propose such a threshold that would give suitable warning of a debris flow. The interpretation and correlation of measured data is still an unsolved problem.

Huang et al. pointed out that FBG-based ground vibration and noise monitoring can be an effective approach for detecting the occurrence of landslide and debris flows [64]. The Mach-Zehnder and Sagnac hybrid interferometer were developed to establish the sensing system. The benefit of this approach is that the FBG signals can be transmitted to longer distance without affecting the sensitivity than conventional systems such as geophones. They conducted a field trial in a debris flow site in Taiwan. The fiber optic vibration sensing system is found to be effective for sensing ground vibration between 10 and 200 Hz frequency range.

### 4.3. Land Subsidence Monitoring

**Figure 10:**
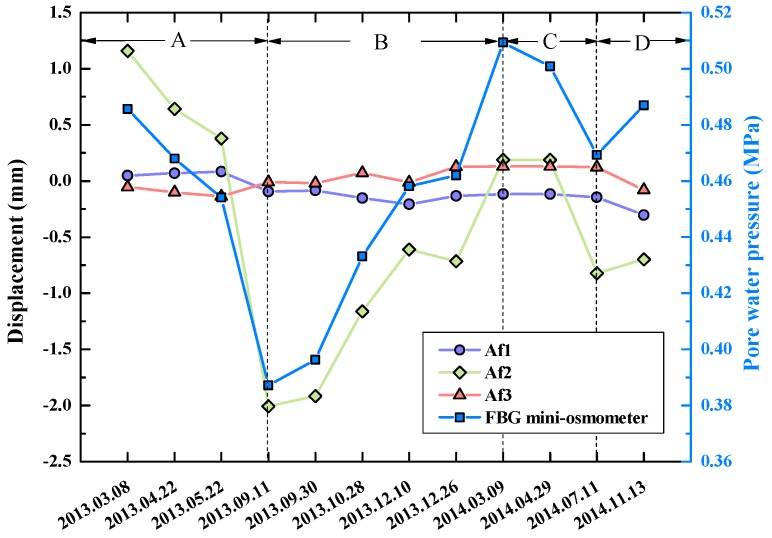
Monitoring results of displacement and pore water pressure during the field test [56]. Af: Aquifer.

Due to rapid growths of population and industry demand in developing countries, large amount of groundwater is being consumed in urban areas. Due to the compaction of susceptible aquifer systems which comprise aquifers and aquitards, serious land subsidence is induced. In the past few decades, ground-based (e.g., GPS, and Tripod LiDAR) and remotely sensed (e.g., InSAR and LiDAR) geodetic techniques have been employed to measure the movements of land surface [74]. On the contrary, borehole extensometers are utilized to measure vertical displacements. In Japan, fully-distributed FOS systems have been introduced to monitor formation compaction accompanying the exploitation of natural gases in boreholes [75]. Wu et al. initiated a study on applying FBG and BOTDR techniques to land subsidence monitoring in Suzhou, China [56]. There were three aquifers in the study site, namely Af1, Af2 and Af3. The fully distributed strain sensing cables were vertically installed in boreholes for displacement monitoring. The displacements were calculated based on the axial strain measurements of the cables. An FBG mini-osmometer was installed in the borehole for pore water pressure monitoring. As shown in Figure 10, the change of pore water pressure had a close relationship with the displacement of Af3. It is seen that the pore water pressure decreased during period A and increased during period B, then re-decreased during period C and re-increased during period D. This was attributed to the larger consumption of groundwater in the summer than in the winter.

Groundwater exploitation, along with coal mining, can also cause deformations of overlying rock strata and fracture of shaft linings, which seriously affect the safe production of coal mine. In this aspect, experimental observations and theoretical calculations indicated that the FBG-based monitoring system was more sensitive to tensile strain than compressive tensile strain for rock layers under high loads [76]. Lately, Chai et al. installed FBG sensors into a deep borehole to continuously measure the deformation of rock strata and shaft linings [33]. The monitoring data could help determine the health condition of shaft linings, thus ensuring the safety of coal mining.

Besides groundwater exploitation, construction activities also induce ground settlement. With the advances of highway transportation, widening or reconstruction of road embankments has been encountered from time to time. Differential settlements become serious if the fill materials of the new embankment are not well compacted. Weng et al. employed the quasi-distributed FBG strain sensors to monitor the pavement behavior after embankment widening [77,78]. In their full-scale tests, 120 FBG sensors were embedded in the asphalt concrete pavement in the longitudinal and transverse directions. Then differential settlements were applied using a subsidence simulation system consisting of 138 hydraulic jacks. The monitoring results clearly show that the geogrid reinforcement in the embankment enhanced the structural integrity of the pavement. The strains of the base pavement layer were considerably larger than those of the upper layers.

**Figure 11:**
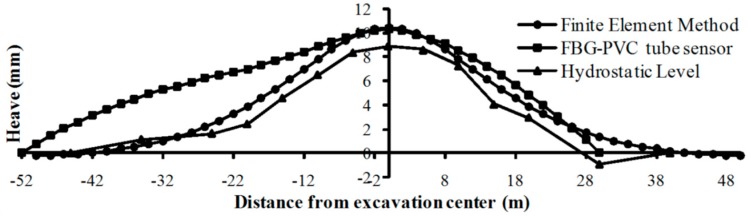
Comparison of tunnel heaves from finite element analysis, FBG sensors and Hydrostatic Level [79].

In large cities all over the world, underground metro tunneling and excavation have resulted in significant ground settlements, which may endanger adjacent buildings and geo-structures. Wang et al. introduced a first attempt to use a series of FBG sensors to measure the tunnel deformation during overhead excavation [79]. The FBG-PVC tube sensors with a total length of 84 m were installed in a shield tunnel of Shanghai Metro Line 2. The deformation profiles were calculated using the theory of bending beam, which agreed well with the Hydrostatic Level readings and finite element analysis results (Figure 11).

### 4.4. Earth Fissure, Fault and Soil Cracking Monitoring

**Figure 12:**
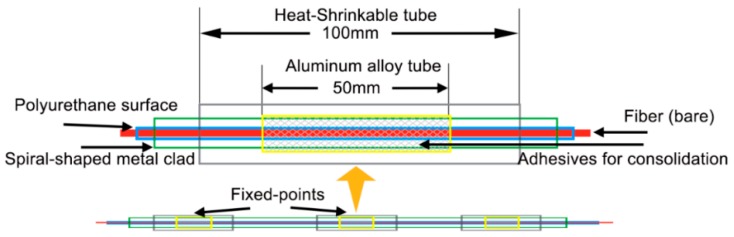
Fixed-point distributed optical fiber sensor for earth fissure monitoring.

Earth fissure is a geological phenomenon which is often closely related to land subsidence caused by engineering activities, especially the excessive exploitation of groundwater. Leveling Survey, photo-geological analysis, crackmeters, tape extensometers, GPS and InSAR have been extensively deployed to monitor earth fissure hazards all around the world [80]. Lu et al. investigated the feasibility of using fixed-point distributed optical fiber sensor in earth fissure monitoring [30]. Both FBG and BOTDR fibers were embedded in aluminum tubes with equally spaced fixed points (Figure 12). In this way, they became long gauge displacement sensors. This installation method can provide the fiber with better protection and the measuring accuracy and ranges of cracking width are adjustable according to specific instrumentation requirements. The sensor system was successfully used in an earth fissure site in Wuxi, China for capturing the trends of crack opening.

Zhang et al. investigated the feasibility of crack detection in expensive soil using embedded FBG strain sensors [81]. They performed a one-dimensional simulation test in laboratory and successfully captured the whole process of soil shrinkage and cracking due to dehydration. The tempo-spatial strain distributions and strain rates clearly show that before crack occured, soil was subjected to shrinkage when the water content reduced. The strains at the desiccation crack location converted from compressive to tensile and slippage occured at the soil-fiber interface if the overburden pressure was very low.

Another interesting applicaiton was reported by Schmidt-Hattenberger et al. for using FBG sensors to monitor large fault zones [82]. The sensors were installed in a research borehole in Greece for long-term monitoring of strains and stresses in active geologic faults.

## 5. Challenges and Future Trends

Various research has proved the feasibility and reliability of FBG sensing systems for field monitoring of geohazards. However, we should know the FBG sensors are not universally effective. First, the FBG sensors can only perform quasi-distributed monitoring and the sensor number in series is limited. Fully distributed sensing technologies, such as BOTDR and Rayleigh backscattering optical frequency domain reflectometry (OFDR) have advantages over FBG in measured length and the amount of information. Hence, these technologies are employed mostly for long-distance and large-magnitude monitoring of geohazards which allows for low measurement accuracy and frequency. Comparatively, FBG is more suitable to be incorporated into geotechnical instrumentation such as an inclinometer for accurate and real-time measurement. On the other hand, the cost of an FBG system is still higher than conventional ones [83]. For instance, the prices of an interrogator and a FBG sensor are about £20,000 and £50–300, respectively. But it is predictable that once the FBG system becomes common practice, the cost will decrease significantly [4].

In general, FBG and conventional sensor systems, especially the remote monitoring systems, are complementary and both approaches are of interest. The field performance of FBG sensors should be validated using conventional ones. However, only a few cases of integrated monitoring of geohazards using both technologies have been reported before. Advanced monitoring methods, appropriate layout of different types of sensors, and a smart warning system are needed for practical monitoring and prediction of geohazards such as landslides. Based on the above review, we propose that the following seven trends in FBG-based geohazards monitoring will be realized in the future.

(1) Development of cost-effective fiber optic demodulation and integration technique. For field applications, rugged and portable devices with low power consumption are desired, so that continuous measurements can be conducted in all-weather conditions. In the future, the solar energy power supply system and general packet radio service (GPRS)-based wireless data transmission will be used to establish the next-generation remote and unattended monitoring system. Monitoring data will be directly transmitted to host computers and then uploaded on websites accessible to interested parties. In addition, hundreds of FBG sensors will be multiplexed in series along a fiber using new demodulation techniques;
(2) Development of innovative sensors for capturing three-dimensional strain and stress states in complicated geological structures, e.g., soil strain rosettes and total stress cells. Because extreme conditions may be encountered during monitoring, robust sensors for large deformation, high temperature and pressure measurements will be developed. Most importantly, these sensors should be correctly calibrated prior to operation;
(3) Development of self-temperature compensation method. Using special algorithm, the discrimination of temperature and strain effect can be obtained. Alternatively, the Bragg wavelength shift will not be influenced by temperature change with the help of new sensor design;
(4) Development of convenient packaging and protection methods. For instance, adaptive materials such as basalt fiber-reinforced polymers can be used. One advantage of this material is that such polymers are made from natural basalt rocks. Using special sensor packaging methods, the increase or decrease of measurement sensitivity can be achieved;
(5) Realization of real-time process, error analysis and abnormality recognition of huge monitoring data. A smart monitoring system should have a powerful data management subsystem with the functions of automated visualization, self-diagnosis, and self-repair. To efficiently process and interpret the huge data retrieved from FBG sensors, the integration of the techniques of big data digging and internet of things is preferred. This is expected to be a hot research topic in the near future;
(6) Establishment of theories for forecasting and early warning of abrupt and progressive geohazards. There have been numerous theories of geomechanical analyses, geohazards evaluation and risk assessment. How to relate the field monitoring data to these theories is an urgent task to solve. The Bayesian updating methodology may be a promising tool in this respect [84,85];
(7) Establishment of guidelines and standards for system deployment and maintenance, especially for harsh monitoring geoenvironments [86]. Standardization of the instrumentation program is key to future advances of FBG monitoring systems. Although abundant knowledge and experience have been gained, for those involved in the instrumentation works, a lack of technical instructions will result in unreliable or unavailable field monitoring data. In the future, the field instrumentation team should be well educated and experienced. Only in this way, can the FBG-based geohazard monitoring technology achieve the full market potential.

## 6. Conclusions

This paper presents an up-to-date review of research and development activities in geohazard monitoring using FBG sensing technologies. The principle of the FBG sensing technology has been introduced briefly, and a comparison of features, including measurement accuracy and distance resolution, is given. Due to the inherent advantageous properties of FBG, such as immunity to EMI, being waterproof, and resistance to corrosion, FBG sensing systems have emerged as a suitable solution in monitoring and early warning of geohazards. As FBG enables quasi-distributed, automated, and long-distance geotechnical monitoring, it has great potential to replace point-mode monitoring techniques. At the same time, the FBG systems can greatly improve monitoring precision and substantially improve geohazard mitigation levels.

Currently, the application of FBG systems for long-term monitoring and early warning of geohazards is still in its infancy and has not been widely recognized. Past experiences, including those gained in this review, tell us that close collaboration of a group of geotechnical practitioners and optoelectronic engineers plays a vital role in the successful implementation of fiber optic geohazard monitoring.
